# Experiment Investigation of the Compression Behaviors of Nickel-Coated Hybrid Lattice Structure with Enhanced Mechanical Properties

**DOI:** 10.3390/mi14101959

**Published:** 2023-10-21

**Authors:** Xiuxia Geng, Mingzhi Wang, Bingyu Hou

**Affiliations:** 1 School of Mechano-Electronic Engineering, Xidian University, Xi’an 710071, China; 2 CityU-Xidian Joint Laboratory of Micro/Nano Manufacturing, Shenzhen 518057, China

**Keywords:** microlattice, additive manufacturing, nickel plating, mechanical properties, energy absorption capacity

## Abstract

The lattice metamaterial has attracted extensive attention due to its excellent specific strength, energy absorption capacity, and strong designability of the cell structure. This paper aims to explore the functional nickel plating on the basis of biomimetic-designed lattice structures, in order to achieve higher stiffness, strength, and energy absorption characteristics. Two typical structures, the body-centered cubic (BCC) lattice and the bioinspired hierarchical circular lattice (HCirC), were considered. The BCC and HCirC lattice templates were prepared based on DLP (digital light processing) 3D printing. Based on this, chemical plating, as well as the composite plating of chemical plating followed by electroplating, was carried out to prepare the corresponding nickel-plated lattice structures. The mechanical properties and deformation failure mechanisms of the resin-based lattice, chemically plated lattice, and composite electroplated lattice structures were studied by using compression experiments. The results show that the metal coating can significantly improve the mechanical properties and energy absorption capacity of microlattices. For example, for the HCirC structure with the loading direction along the *x*-axis, the specific strength, specific stiffness, and specific energy absorption after composite electroplating increased by 546.9%, 120.7%, and 2113.8%, respectively. The shell–core structure formed through composite electroplating is the main factor for improving the mechanical properties of the lattice metamaterial. In addition, the functional nickel plating based on biomimetic structure design can further enhance the improvement space of mechanical performance. The research in this paper provides insights for exploring lighter and stronger lattice metamaterials and their multifunctional applications.

## 1. Introduction

The design and manufacture of micro/nano-structural materials that possess both lightweight and excellent mechanical properties have been a long-standing issue for industrial applications [1,2,3,4]. The microlattice metamaterials have advantages such as high strength, low density, high energy absorption, and strong designability [5,6,7,8], and they have great potential for application in aerospace, transportation, and biomedical fields [9,10,11]. In the past few decades, the development of additive manufacturing technology has promoted the extensive research on microlattice structures. Many lattice structures with excellent mechanical properties, such as body-centered cubic (BCC), octet-truss (OT), and rhombic dodecahedron (RD), have been studied and their mechanical properties were optimized, in order to be used in different engineering fields [12,13,14,15]. With the rapid development of advanced manufacturing technology, the development of engineering materials has shown obvious trends toward light weight, integration, functionalization, and biomimicry [16]. Therefore, achieving enhanced mechanical performance through lattice design and manufacturing is an important research topic for practical engineering application.

After millions of years of evolution, the microstructure of biological materials has come to exhibit extremely tough and strong mechanical properties far superior to those of traditional artificial materials [17,18]. Inspired by natural organisms, many novel structures with excellent mechanical performance have been proposed, such as the wall-septa sandwich structure inspired by squid bone [19,20], the Bouligand structure inspired by exoskeletons of crustaceans [21,22], the bio-inspired hierarchical and tree-like multi-cell tubular (BHMB) structure proposed based on natural structures like giant lotus leaves [18], and the graded porous structure designed by mimicking the microstructure of butterfly wings [23]. The hierarchical designs inspired by nature are important strategies to enhance the mechanical performance of lattice structures. In many biological structures, such as sponge frameworks, bamboo, wood, shells, and wheat [24,25], hierarchical microstructures are commonly found. The superior mechanical performance of these biomaterials are attributed to the inherent hierarchical structure [12], which plays an essential role in achieving the trade-off between strength and density. Dong et al. [26] studied the compression deformation of self-similar layered octet-truss structures. They found that the hierarchical structures exhibit highly competitive performance compared to other cellular materials. Wang et al. [27] proposed a novel hierarchical face-centered cubic (HFCC) lattice design, which possesses an increased number of plastic hinge joints and significantly enhanced energy absorption performance. This provided an effective and feasible approach for investigating the energy absorbers with high-performance. Similarly, designs of embedded FCC-graded lattice structures have been proposed, allowing customizable stress platforms, which have important implications for multi-scenario applications and the engineering design of lattice structures. Their studies demonstrate that the biomimetic hierarchical strategy is helpful to achieve a unique performance, which usually cannot be attained with conventional materials. In our previous research, we employed biomimetic hierarchical strategies to design lattice structures, e.g., HSCBCC [28] and HCirC [29], with internal hierarchical structures. These novel structures have also exhibited excellent mechanical performance and energy absorption capacity. To further explore the structural advantages of biomimetic hierarchical lattice structures, metal coating processes were considered to fabricate metal microlattice structures and investigate their mechanical properties.

The metal microlattice structure not only possesses excellent performance but also has special properties such as conductivity, thermal conduction, and corrosion resistance. It has a wide range of applications in various industries such as aerospace, automotive, and biomedicine [30,31]. The surface coating techniques for manufacturing metal microlattice structures mainly include electroless plating [32], electroplating [33], sputtering deposition [34], vacuum metallization [35], and so on. Among them, electroless plating is widely used in the early research of microlattice surface coating, due to its inexpensive equipment, lack of surface morphology restrictions, low cost, and reliable large-area deposition capabilities [36]. In 2011, Schaedler et al. used electroless nickel plating to prepare the lightest material at that time—metal microlattice [37]. This interesting discovery has opened the door to the study of various types of coatings and truss arrangements, including periodic structured metals such as square honeycombs, diamond cores, and octet truss lattices [38].

The general steps for metal microlattice coatings are as follows: first, use 3D printing technology to prepare a polymer template, and then coat the metal on the surface of the microlattice template. For example, Torrents et al. [31,39] obtained templates using self-propagating light-wave technology and then manufactured nickel hollow microlattice structures using both electroless plating and electroplating. In 2016, Zheng et al. [40] made a breakthrough by using high-resolution large-scale additive manufacturing technology to produce microlattice metal structures spanning seven orders of magnitude. However, the prototypes were very small and there is still a long way to go before practical use. Lee et al. [41] used radio-frequency magnetron sputtering to simultaneously coat copper and zirconium on the polymer surface, and after the sputtering deposition, used focused ion beam milling to polish the surfaces of the cube specimens, exposing the polymer skeleton underneath the coating, before removing the skeleton in an oxygen plasma etcher. Metal microlattice structures manufactured using this method typically have micro- and nanoscale features and low-density ultra-light structures. In 2018, Gao et al. [42] applied the “smaller is stronger” characteristic of the nanoscale to the microscale materials by using high-entropy alloy thin films (HEA). Based on high-precision two-photon polymerization 3D printing technology combined with physical vapor deposition, they successfully prepared metal microlattice structures with a minimum feature size of only 500 nm. Gao et al. [43] also obtained templates using two-photon laser lithography, and then used sputtering to obtain a 10 nm thick gold layer. Xu et al. [33] first used sputtering to deposit a layer of Au coating on the polymer skeleton, and then used electroplating to thicken the Au layer to 5 μm. Fan et al. [44] used electroless plating to coat Ni-P-diamond on diamond microlattice structures, demonstrating that the incorporation of diamond particles in the metal coating can effectively enhance the mechanical performance of the microlattice. In general, coating the metal on the surface of the microlattice can enhance its mechanical properties such as specific stiffness, specific strength, and energy absorption capacity. Zheng et al. [45] investigated the nickel coating of 3D-printed auxetic metamaterials and their mechanical performance. The compression experiment result showed that the integration of electroless plating and 3D printing enabled controllable conduction and mechanical properties. Xiao et al. [46] explored the prosthesis application of a graded lattice metamaterial, based on the 3D printing of a resin lattice and metallic coating. The mechanical properties of the fabricated lattices were investigated using a compression experiment, and the results showed that the metallic Ti-coated lattices exhibited an increase in strength by a factor of 2–3. The integration of 3D printing and metallic coatings was an effective way to fabricate the lattice metamaterials with enhanced mechanical properties [45,46]. However, although some studies have explored the efficacy of metallic coatings on the performance of microlattice structures, there is still relatively limited research on the mechanisms and performance of metallic coatings on lattice structures with various unit cell configurations.

In the present work, chemical plating and electroplating were performed on 3D-printed resin lattice samples. The conventional BCC and the bioinspired hierarchical circular (HBCC) lattice structures were considered. The Ni-P coating was deposited on the target lattice structures using the chemical composite plating method, and then a Ni metal layer was electroplated on the surface of the chemical plating layer. The influence of the coating on the mechanical structure performance of microlattices was investigated, and the compression failure mechanism of the metal-plated lattice was analyzed. The metal-coated microlattice structures studied in this paper have high mechanical performance and energy absorption capacity. Additionally, they possess advantages in corrosion resistance, which will enhance the service life of aerospace components.

## 2. Lattice Design and Nickel Coating Experiment

### 2.1. Lattice Structures

**Figure 1:**
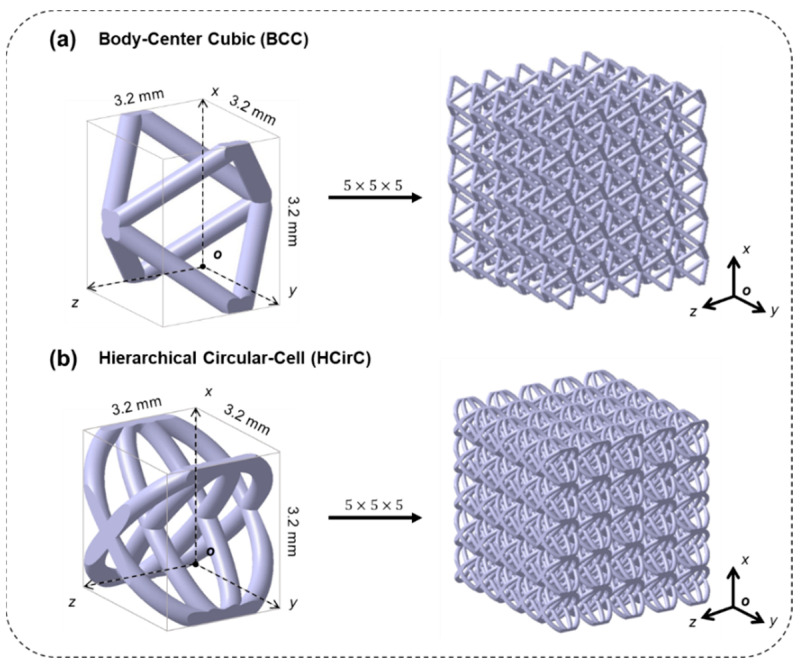
The geometry design of the lattice structures: (**a**) BCC and (**b**) the bio-inspired hierarchical circular-cell lattice structure (HCirC).

In order to investigate the effect of metal coating and topological structure on the mechanical properties of lattice structures, two different lattice structures were considered in the experiments. The selected lattice structures were the traditional body-centered cubic lattice structure (BCC) and the bioinspired hierarchical circular lattice (HCirC) structure, as shown in Figure 1a,b, respectively. The HCirC structure is a bio-inspired hierarchical lattice structure, which has been reported in our previous research [29]. This structure is based on a single-layer circular structure (CirC) and adds internally crossed orthogonal elliptical rings. All the samples used in the experiment have the same unit cell size of 3.2 mm and a rod diameter of 0.4 mm. The lattice structures are composed of a 5 × 5 × 5 array of basic unit cells along the *x*, *y*, and *z* directions, and the geometric coordinates are defined in Figure 1, according to the right-hand principle.

### 2.2. The Nickel Coating Experiment Process

**Figure 2:**
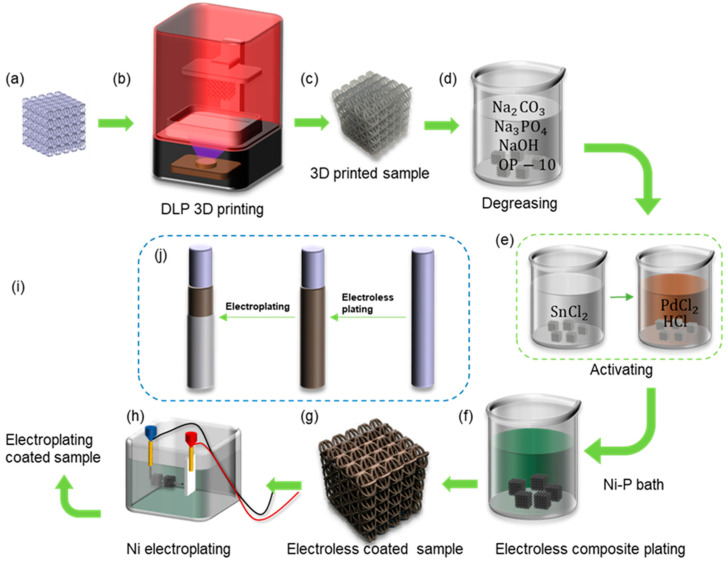
The fabrication processes of the nickel-coated hybrid lattice structure: (**a**) CAD model; (**b**) DLP 3D printing; (**c**) the printed lattice sample; (**d**) degreasing; (**e**) activating; (**f**) Ni-P bath for electroless plating; (**g**) electroless coated sample; (**h**) Ni electroplating; (**i**) the fabricated nickel-coated hybrid lattice sample; (**j**) the electroless plating and electroplating of nickel.

The commercial 3D printer (NOVA 3D Whale 2, manufactured by Shenzhen nova company, Shenzhen, China) based on digital light processing (DLP) technology was used to manufacture the lattice samples. Figure 2a shows the designed CAD models of lattice structures, and Figure 2b shows the 3D printer. Figure 2c shows the resin lattice sample. The procedures for fabricating the shell–core lattice structures with metallic coatings are shown in Figure 2a–j. The geometry of the lattice structure is sliced and imported into the printer system for fabrication. The printer can achieve a minimum printing layer thickness of 0.02–0.15 mm, with a horizontal accuracy of 0.05 mm. After the resin model is printed, it is cleaned with isopropyl alcohol and cured using UV light for 30 min at 60 °C. The post-cured resin-based lattice structure is then used as a template for further chemical and electroplating processes. This forms the resin–metal shell–core lattice structure, with the internal resin-based structure and external metallic coating [36].

In the metallic coating experiment, nickel metal was considered. The coating process involved a combination of chemical and electroplating methods. The main route, as shown in Figure 2j, involved depositing a Ni-P alloy on the surface of the resin substrate using chemical plating, followed by electroplating a layer of pure nickel on top of the chemical plating, to achieve a more continuous, stronger, and evenly distributed coating. Figure 2f shows the preparation of conductive resin–metal microlattice structures using chemical plating, which provided the basic conditions for subsequent electroplating experiments on microlattice structures.

The pre-treatment before electroplating consisted of two steps: degreasing and activation. Degreasing aimed to remove oil and impurities from the surface of the skeleton to facilitate the adsorption of colloidal palladium. Activation refers to attaching chemically catalytic substances to the surface of the plating, enabling the plating to initiate redox reactions. The degreased component was immersed in a solution containing a catalyst with chemical activity such as palladium compound, allowing the formation of a surface layer of catalytic precious metal particles on the resin component [36]. The specific experimental steps and the preparation of chemical solutions are shown in Figure 2. Firstly, the BCC and HCirC resin lattice substrates were placed in the prepared degreasing solution, ultrasonically cleaned, and then rinsed with deionized water. The ingredients and concentrations in the degreasing solution were as follows, anhydrous sodium carbonate (Na_2_CO_3_) 30 g/L, anhydrous trisodium phosphate (Na_3_PO_4_) 50 g/L, sodium hydroxide (NaOH) 25 g/L, and emulsifier OP-10 (C_32_H_58_O_10_) 5 g/L, and the rest was deionized water. The degreasing time was 10 min, and the temperature was maintained at 50 °C. In the next stage, the activation of the sample, as shown in Figure 2e, was carried out by immersing the dried sample in a 10 g/L SnCl_2_ solution for 20 min, followed by rinsing and drying with deionized water and a drying oven. The dried sample was then immersed in a mixture of 0.25 g/L palladium chloride solution and 10 mL/L hydrochloric acid solution for 20 min, with the temperature maintained at around 40 °C.

The prepared nickel plating solution was heated to around 50 °C, and the dried component after activation was placed in the plating solution and further heated. The temperature of the plating solution was kept between 70 and 90 °C, and the pH value was maintained at 4.4–4.8 until the plating reaction was complete. The nickel plating bath contained 30 g/L NiSO_4_∙6(H_2_O), 10 g/L NaH_2_PO_2_, and 50 g/L CH_3_COONa. The technique of nickel chemical plating is very common, and similar coating methods can be seen in many previous works [32,44,45]. After plating, a batch of samples was taken for subsequent electroplating experiments. The experimental steps for electroplating, as shown in Figure 2h, were described as follows: Firstly, the samples after chemical plating were cleaned and dried, and then placed in the electroplating solution. Nickel plates were placed on both sides of the plating tank and immersed in the electroplating solution. The electroplating solution contained 240 g/L NiSO_4_, 45 g/L H_3_BO_3_, 0.7 mL/L C_3_H_5_NaO_3_S, and the pH value was maintained at 4.0–4.5. The nickel plates were used as the anode connected to the positive pole of the power supply, while the plated components served as the cathode connected to the negative pole of the power supply. The thickness of the plating layer could be controlled by adjusting the current density and electroplating time.

### 2.3. Nickel-Coated Lattice Samples

**Figure 3:**
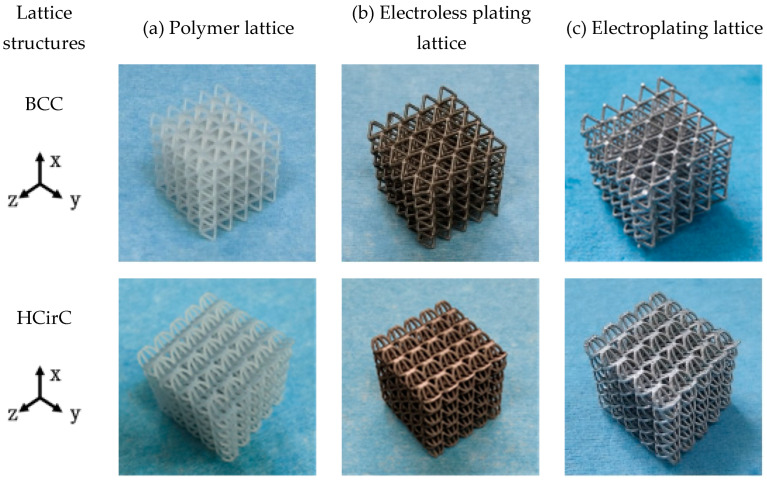
Comparison of the fabricated BCC and HCirC lattice samples before and after coating: (**a**) resin lattice sample; (**b**) after electroless plating; (**c**) after electroplating.

After the chemical plating and electroplating, the lattice samples were cleaned and dried. The 3D-printed samples of BCC and HCirC lattice structures, as well as the samples after chemical plating and electroplating, are shown in Figure 3. Due to the symmetry of the geometric structure, the BCC lattice exhibits isotropic mechanical properties along three orthogonal directions. Therefore, the compressive mechanical properties of the BCC lattice can be characterized by loading along one single direction. The bio-inspired hierarchical HCirC structure is anisotropic, and it requires the loading in two directions to fully quantify its compressive mechanical properties. Figure 3 shows the resin lattice and the chemical plating and electroplating lattice samples of BCC and HCirC lattice structures. From the figures, it can be observed that after chemical plating, the structure appears dark, while after electroplating, the samples exhibit a bright silver-white color. In this study, the Ni coating was considered in the experiment. This is because the Ni coating is widely adopted in fabricating Ni-coated lattice structures. In addition, the Ni thin film is relatively easy to fabricate, and it possesses good stiffness, ductile behaviors, and corrosion resistance. The other thin-film coatings, e.g., Cu, Co, Cr, might be useful in different application situations, e.g., Cu coating possesses good conductive properties, and this will be considered in our future work.

In this study, to further reveal the comparison of the mechanical properties of the lattice structures before and after the two coating methods, the samples before and after the experiments were weighed using a high-precision electronic balance. The mass values were measured from three independent samples for each lattice type and coating method, and the results are listed in Table 1. In addition, the nickel on the surface of the lattice structure was calculated, and their values are also listed in Table 1.

**Figure 4:**
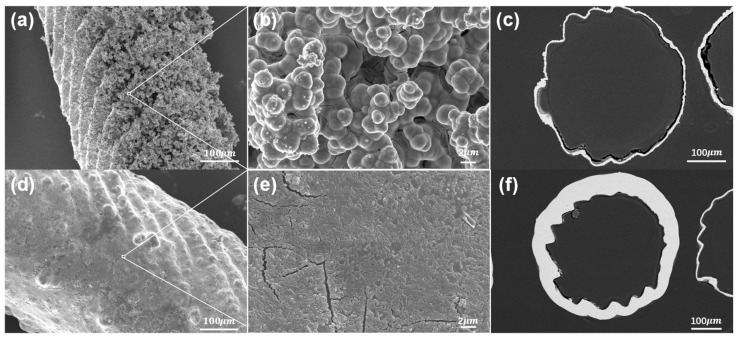
Surface and cross-sectional morphologies of Ni-P composite coating of electroless plating and Ni coating electroplating: (**a**,**b**) SEM morphology of electroless plating surface; (**c**) SEM image of the thickness of the electroless coatings; (**d**,**e**) SEM morphology of electroplating surface; (**f**) SEM image of the thickness of the electroplated coatings.

The surface morphology and microstructure of the samples after nickel plating were studied using scanning electron microscopy (SEM). Figure 4 shows the morphology of the prepared nickel-coated microlattices (operating voltage 10 kV) and the thickness of the surface coating (operating voltage 20 kV), with a scale included at the bottom to represent the size of all relevant features and demonstrate the hierarchical structure. In Figure 4a–c, the surface morphology and thickness of the chemical plating can be observed. The Ni-P coating and the Ni metal obtained on the surface of the microlattices are both uniformly dense and well bonded at the interface. The chemical plating induces the deposition of numerous grains and cells on the surface, which is attributed to the Ni-P thin film deposited through chemical plating. The thickness of the chemical plating can be determined by measuring the width of the nickel layer in the SEM images, as shown in Figure 4c. The thickness of the chemical plating is relatively uniform, ranging from 7 μm to 10 μm. This is consistent with the previously reported characteristics of Ni-P binary chemical deposition [47,48]. Figure 4d,e depict the finer grains on the surface of the electroplated coating, with nickel particles uniformly covering the resin sample surface, resulting in better surface smoothness. As shown in Figure 4f, after electroplating, the surface of the strut becomes smoother and the thickness of the metal coating increases to 22–28 μm.

### 2.4. Compression Experiment

**Figure 5:**
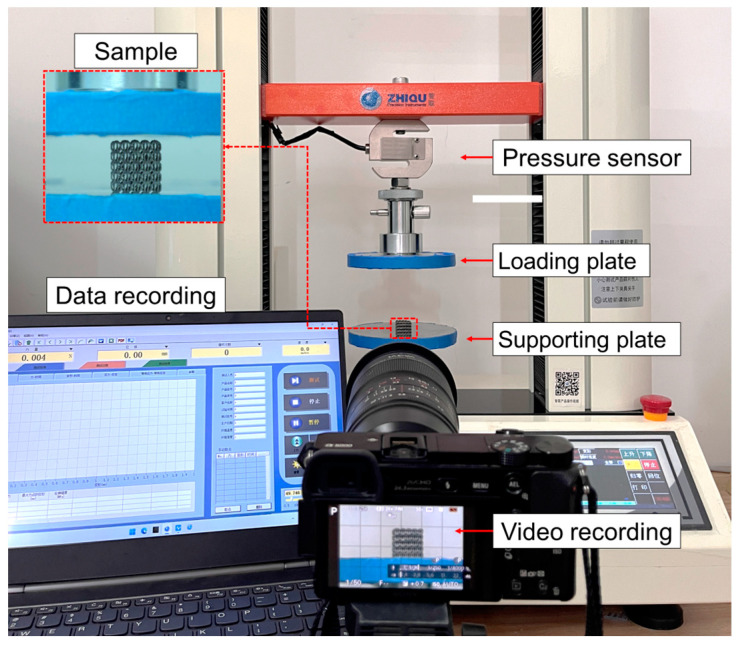
The in situ compression experiment platform.

The quasi-static compression test was conducted using a universal testing machine (ZQ-950B, manufactured by Dongguan zhiqu company, Dongguan, China) with a maximum load of 5 KN at room temperature. Figure 5 shows the experiment platform. The specimen was placed between two parallel plates. During the experiment, the loading speed was set at 3 mm/min until the test reached densification (80% strain), and then stopped. The surfaces of the plates were lubricated to reduce friction and prevent lateral deformation. The compression stress–strain curves were obtained from the load–displacement during the compression experiments. The deformation behaviors of the lattice structures under axial compression were recorded using a high-resolution camera.

## 3. Results and Discussion

### 3.1. Compression Mechanical Properties of the Lattice Structures

**Figure 6:**
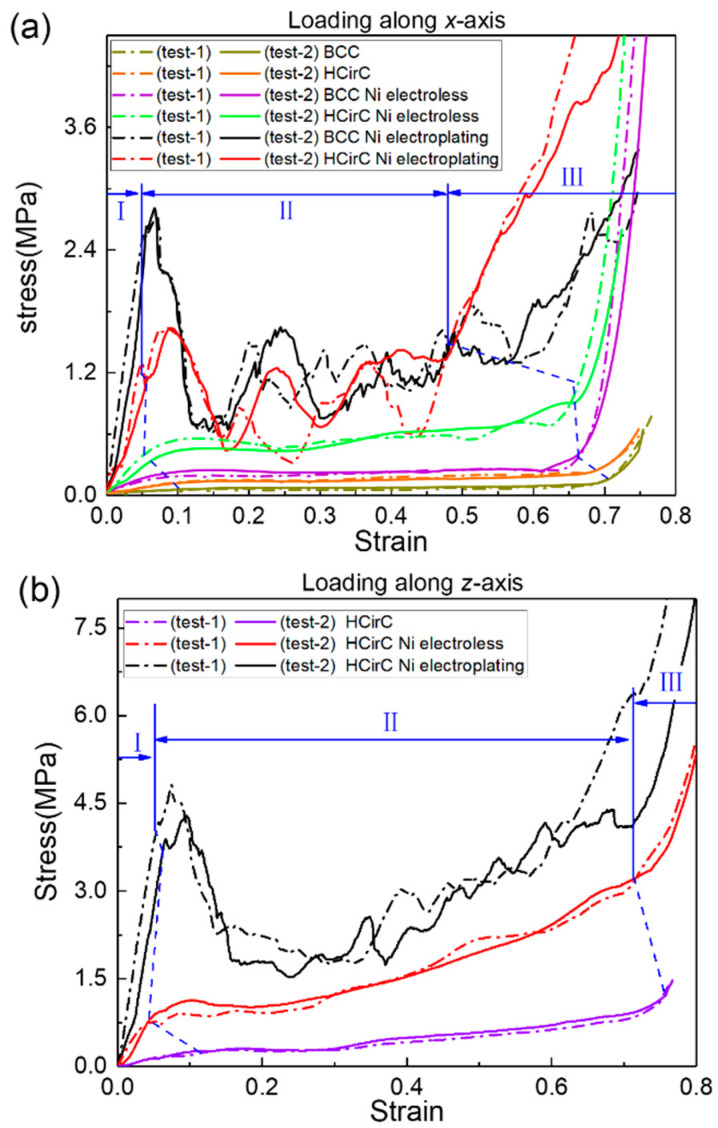
Comparison of the compression stress–strain curves of the BCC and HCirC lattice structures, and here I–III represents the different deformation stages: (**a**) loading direction along the *x*-axis; (**b**) loading direction along the *z*-axis.

The axial compression experiments were performed, and the compressive curves of BCC and HCirC before coating, after electroless plating, and after electroplating were compared. Figure 6a shows the compression stress curves along the *x*-loading direction before and after coating for BCC and HCirC structures, and Figure 6b shows the compression stress curves along the loading direction *z*-axis before and after coating for HCirC structures. In Figure 6, the compression curves obtained from two independent experiments, for each lattice type, are plotted together, and results indicate the good repeatability of the experiments. Those compression curves were used to calculate the stiffness, strength, and energy absorption capacity. In this study, two independent experiments were performed, and the averaged data values were extracted from the experiments. So, the measured data essentially represent the statistical averaged values.

In Figure 6, the BCC and HCirC lattice structures and the compression curves after the metal coating show three distinct stages. They are the initial elastic, yielding and plastic, and densification stages. In the first stage, the compression stress linearly increases with the increase in strain, and the yielding behavior occurs. In the second stage, under larger compression strain, the lattice exhibits obvious progressive failure and collapse. In the third stage, the rods of the lattice structure come into contact with each other, resulting in the rapid increase in compression stress.

It is worth noting, as shown in Figure 6a, that when loaded along the *x*-direction, both BCC and HCirC exhibit relatively smooth compression curves. Additionally, in this situation, no significant initial peak stress is observed. After chemical plating, both the BCC and HCirC show higher stiffness in the initial elastic stage. The lattice structure exhibits relatively smooth compression curves without any obvious initial peak stress. That is to say, the deformation of the resin and chemical plating lattice structures tends to be bending-dominated, when loading along the *x*-axis. For the BCC structure, chemical plating causes the densification stage to shift from a strain of 0.673 to 0.611; for the HCirC structure, chemical plating causes the densification stage to shift from a strain of 0.705 to 0.697; and after the electroplating, the stiffness of both the BCC and HCirC structures significantly increases in the first stage. In the yield and plastic stages, obvious initial peak stresses and stress oscillations can be observed, which indicates that the deformation and collapse mechanism of the electroplating structure is stretching-dominated.

Figure 6b shows the stress–strain curve of the HCirC along the loading direction *z*-axis. Similarly, the stress–strain curve of the HCirC structure is relatively smooth, without any obvious initial peak stress. After chemical plating, the HCirC structure exhibits a noticeable increase in stiffness and plateau stress. Similarly, electroplating causes the HCirC structure to exhibit obvious initial peak stress and stress fluctuations, leading to an earlier onset of the densification stage.

Therefore, the chemical plating can enhance the stiffness and plateau stress of both the BCC and HCirC, and the stress–strain curves exhibit a smooth transition from elastic deformation to plastic deformation during the plateau stage, as well as a stable stress response. However, after electroplating (with respect to the resin samples), both the BCC and HCirC show significant oscillations in the stress–strain curves, indicating a change of the deformation and collapse mechanism, from bending- to stretching-dominated behaviors.

Both the BCC and HCirC lattice structures exhibit higher plateau stress in the compression curves after the chemical plating experiment, but the enhancement effect is not significant. However, when the composite electroplating is adopted, the enhancement effect becomes very obvious. This phenomenon can be explained by the fact that when the chemical plating is used, the coating thickness is relatively thin, e.g., about 7 μm to 10 μm, and the deformation and compression responses are mainly dominated by the resin lattice substrates. When the lattice structure experienced the further electroplating, the thickness of the coating is thicker, e.g., the thickness value reaches 22 μm to 28 μm, and then the deformation and mechanical response of the electroplating lattice samples are mainly dominated by the combined effect of the shell–core structures. This explains why the obvious increase in the mechanical properties and the change of deformation mechanism can be observed. That is to say, the metallic coating was the main reason that caused the change of deformation mechanism and deformation mode, which finally caused the change in the stress–strain curve. Therefore, the shell–core structure formed by the metal composite coating is beneficial for enhancing the mechanical properties and energy absorption of the lattice structures.

**Figure 7:**
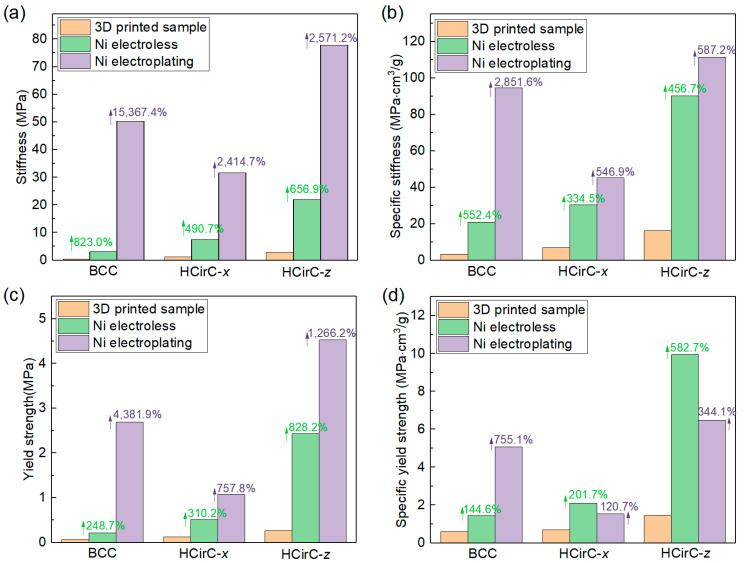
Comparison of the mechanical properties of lattice structures: (**a**) stiffness; (**b**) yield stress; (**c**) specific stiffness; and (**d**) specific yield strength.

The mechanical properties of the BCC and HCirC lattice structures before and after plating are obtained from the uniaxial experiment curves. Table 2 lists the results of the mechanical properties. The specific mechanical values (e.g., stiffness and strength) are calculated by dividing their corresponding relative density. Figure 7 shows the comparison of the mechanical properties of BCC and HcirC before and after nickel-coating.

From Table 2 and Figure 7, it can be observed that the lattice structures exhibit significantly enhanced mechanical properties after chemical plating and electroplating. In terms of the stiffness (Figure 7a), the BCC lattice structure is enhanced by 823.0% and 15,367.4% after chemical plating and electroplating, respectively. The HCirC lattice structure shows enhancements of 490.7% and 2414.7% after chemical plating and electroplating, respectively, when the loading direction is along the *x*-axis. In addition, the HCirC lattice structure shows enhancements of 656.9% and 2571.2% after chemical plating and electroplating, respectively, when the loading direction is along the *z*-axis.

In terms of strength (Figure 7b), the BCC structure is enhanced by 248.7% and 4381.0% after chemical plating and electroplating, respectively. The HCirC lattice structure shows enhancements of 310.2% and 757.8% after chemical plating and electroplating, respectively, when the loading direction is along the *x*-axis. In addition, the HCirC lattice structure shows enhancements of 828.2% and 1626.2% after chemical plating and electroplating, respectively, when the loading direction is along the *z*-axis.

In terms of specific stiffness (Figure 7c), the BCC lattice structure is enhanced by 552.4% and 2851.6% after chemical plating and electroplating, respectively. The HCirC lattice structure shows enhancements of 334.5% and 546.9% after chemical plating and electroplating, respectively, when the loading direction is along the *x*-axis. In addition, the HCirC lattice structure shows enhancements of 456.7% and 587.2% after chemical plating and electroplating, respectively, when the loading direction is along the *z*-axis.

In terms of the specific strength (Figure 7d), the BCC structure is enhanced by 144.6% and 755.1% after chemical plating and electroplating, respectively. The HCirC lattice structure shows enhancements of 201.7% and 120.7% after chemical plating and electroplating, respectively, when the loading direction is along the *x*-axis. In addition, the HCirC lattice structure shows enhancements of 582.7% and 344.1% after chemical plating and electroplating, respectively, when the loading direction is along the *z*-axis.

As can be seen from Figure 7a–d, for the HCirC lattice structures, the enhancement effect of specific strength after electroplating is no stronger than that of chemical plating. From the above data, it can be seen that both electroplating and chemical plating can significantly enhance the mechanical properties of the lattice structures, and the enhancement effect of electroplating is generally better than that of chemical plating alone. Therefore, this further proves that the shell–core structure formed by the metal composite coating is beneficial for enhancing the mechanical properties of lattice structures. In addition, by comparing the performance enhancement of the BCC structure and the bioinspired HCirC structure, it can be seen that under the same loading direction and the same plating method, the HCirC structure exhibits higher overall mechanical properties than the BCC structure does, especially when loaded along the *z*-axis. Thus, it can be seen that by adopting a bioinspired unit cell design and combining the advanced coating technologies, the performance enhancement space of lattice metamaterials can be further improved.

### 3.2. Deformation and Collapse Behaviors

**Figure 8:**
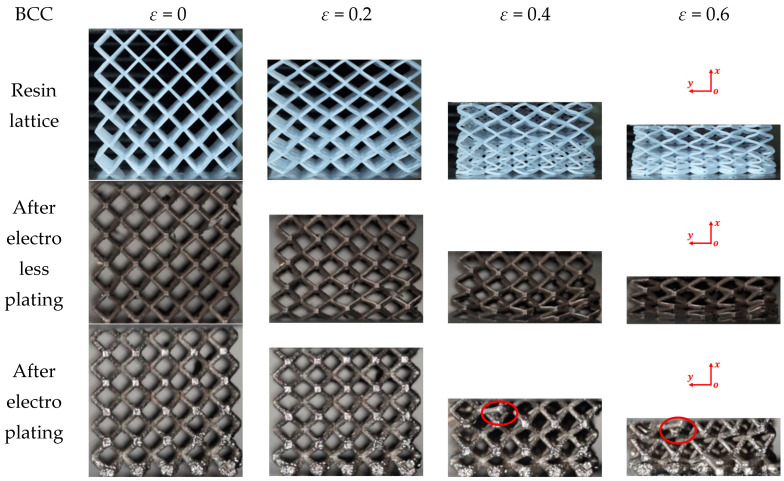
Deformation and failure behaviors of the BCC lattice structure with the loading direction along the *x*-axis (XY view), here the red circle indicates the local collapse and fracture regions of lattice structure.

**Figure 9:**
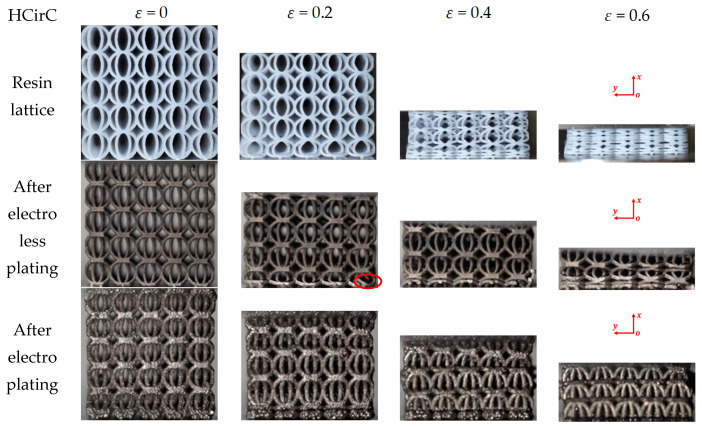
Deformation and failure behaviors of the HCirC lattice structure with the loading direction along the *x*-axis (XY view), here the red circle indicates the local collapse and fracture regions of lattice structure.

**Figure 10:**
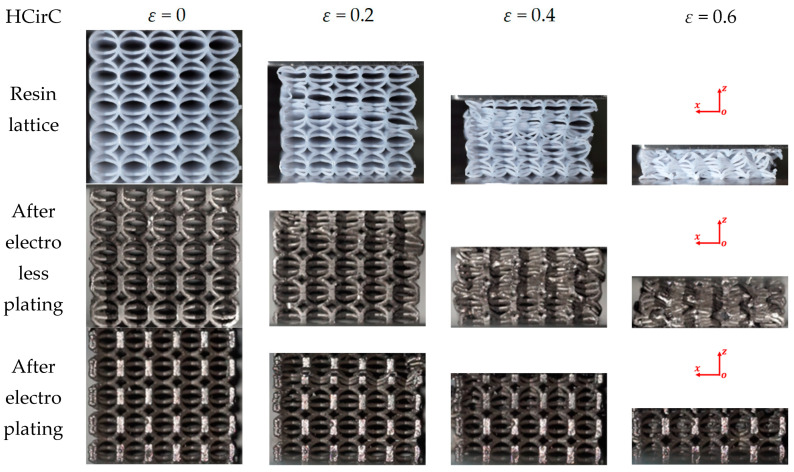
Deformation and failure behaviors of the HCirC lattice structure with the loading direction along *z*-axis (XZ view).

In the compression experiment, the deformations of the lattice structures before and after metal plating were recorded, in order to reveal their deformation mechanisms. The compression behaviors of the resin samples and the samples after chemical plating and electroplating are shown in Figure 8, Figure 9 and Figure 10, respectively, and the deformation snapshots corresponding to strain values of 0, 0.2, 0.4, and 0.6 were plotted for each structure.

The compression and deformation behaviors of the resin samples and the samples after chemical plating and electroplating are shown in Figure 8, Figure 9 and Figure 10, respectively. The BCC lattice structure is isotropic along three orthogonal directions, and only one loading direction is needed, as shown in Figure 8. For the HCirC lattice structure, two loading directions in compression experiments were performed. Figure 9 shows the compression behaviors of the HCirC lattice along the *x*-axis loading direction, and Figure 10 shows the compression behaviors of the HCirC lattice along the *z*-axis loading direction. As can be seen from Figure 8, Figure 9 and Figure 10, the lattice structures undergo three distinct deformation stages, including the initial deformation and buckling stage, the progressive collapse stage under larger compressive strain, and the final densification stage.

In Figure 8, the deformation modes of the BCC resin lattice structure and the chemically plated lattice structure are very similar. In the initial deformation stage (e.g., *ε* = 0.2), both the resin lattice and the chemically plated lattice exhibit relatively uniform deformation, and the lower layers of the structure begin to collapse first, while the top and bottom layers of the electroplated lattice show obvious deformation with the tendency to collapse. With the increase in compression strain (e.g., the strain is larger than 0.4), the rods of the resin lattice and the chemically plated lattice gradually bend around the nodes, and an obvious shear band can be observed. The electroplated lattice structure exhibits the layer-by-layer collapse behaviors, which is consistent with the obvious oscillation of the compression stress shown in Figure 6a.

Figure 9 shows the compression behaviors of HCirC before and after metal plating, and the compression direction is along the *x*-axis. In Figure 9, both the resin and chemically plated HCirC lattice structures exhibit very similar and uniform deformation. For the resin and chemically plated HCirC lattice structures, in the initial stage of compression (e.g., *ε* = 0.2), the local fracture of rods can be observed. There is significant compression deformation in the bottom layer. With the increase in compression strain (e.g., *ε* = 0.4), it can be observed that the bottom layer collapses first. Finally, with the further increase in compression strain (e.g., *ε* = 0.6), the number of collapsed layers gradually increases to three layers. For the electroplated HCirC lattice structure, the deformation process follows a layer-by-layer collapse pattern, which is consistent with the obvious oscillation behaviors of the compression stress in Figure 6a. In the initial stage (e.g., *ε* = 0.2), plastic bending occurs in the upper and lower layers, and rods start to fracture. In Figure 9, it can be seen that each layer of the collapsed structure has incomplete half rings, indicating that the collapse is caused by excessive distortion of the rods around the central node of the cell, leading to rod fracture at the nodes.

Figure 10 shows the compression behaviors of HCirC before and after plating, and the loading direction is along the *z*-axis. As can be seen from Figure 10, both the resin samples and the samples after chemical plating exhibit similar deformation processes. In the initial stage (e.g., *ε* = 0.2), the upper and lower layers undergo deformation and collapse first, and the cells begin to contact with each other, leading to the increase in compression stress. Due to the enhanced mechanical properties of the chemically plated lattice structure, the initial peak stress of the chemically plated lattice structure is higher. With the increase in compressive strain (e.g., *ε* = 0.4), the middle layers of the lattice structure begin to collapse. For the electroplated HCirC lattice structure, in the initial stage (e.g., ε = 0.2), the deformation is concentrated in the second layer, and subsequent layers also start to collapse. With the strain increasing (e.g., *ε* = 0.4), the collapse deformation of the HCirC structure gradually extends to the lower layers. The contact between adjacent layers of rods leads to the progressive increase in the compression stress.

**Figure 11:**
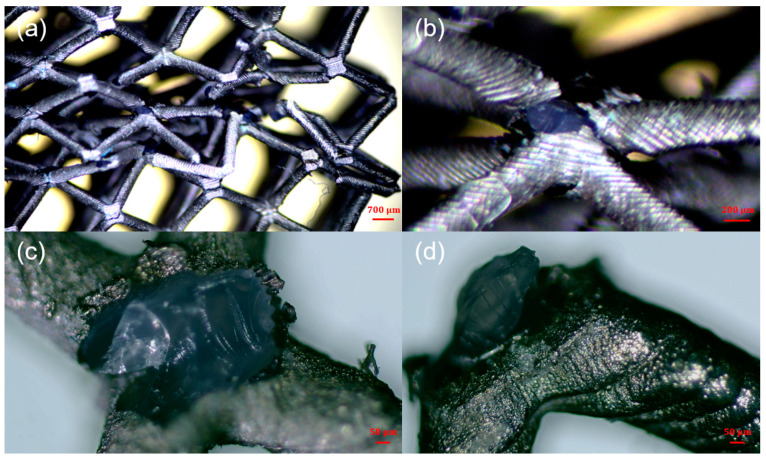
The fracture surface morphologies of the nickel-coated lattice samples: (**a**–**d**) the corresponding microscopic figures at different magnifications.

In Figure 8, Figure 9 and Figure 10, the comparison results of the deformation and failure behaviors of resin samples and the chemically plated and electroplated samples of BCC and HCirC lattice structures indicated that the chemical plating has limited influence on the deformation behaviors of BCC and HCirC lattice structures. This may be because the thickness of the metal coating after chemical plating is very thin, and the deformation behaviors is very similar to the corresponding resin samples. That is to say, the deformation mode of the BCC and HCirC structures after chemical plating is still dominated by their resin substrates, while for the composite plating technique, e.g., electroplating, it has a significant impact on the deformation of the BCC and HCirC lattice structures. In Figure 8, Figure 9 and Figure 10, it can be observed that the electroplated lattice structures collapse layer by layer, leading to distinct stress oscillations. So, the deformation mode changes from bending- to stretching-dominated, and the load-bearing capability increases accordingly. The fracture surface morphologies of the nickel electroplating lattice samples are further shown in Figure 11. Here, for simplicity, only the fracture surface morphologies of the electroplated BCC lattice around a compression strain if 0.4 is shown. In Figure 11, it can be seen that with the deformation and collapse of the lattice structure, the surface nickel coating begins to peel off and fracture from the resin surface, thus exposing the internal resin material. This further reflects that the core–shell structure formed by the coating and resin and the thickness of the nickel coating are the main factors for the enhancement in mechanical properties. Therefore, after the metal electroplating, the mechanical strength of the lattice structures will be significantly enhanced.

### 3.3. Energy Absorption Capacity

The energy absorption is also a very significant indicator for evaluating the mechanical performance of lattice structures [49,50,51]. In particular, the specific energy absorption is defined as the ratio of energy absorption to the total mass of the lattice structure [51], as shown in Equation (1).

(1) $$SEA=\frac{TEA}{M}=\frac{\int_{0}^{U}P_{i}du}{M}$$

where $P_{i}$ represents the load, *U* represents the maximum displacement during the compression experiment, and *M* is the total mass. The total energy absorption (TEA) is defined as the integration of the compressive load and displacement, which can be expressed as $\int_{0}^{U}P_{i}du$ [49,51].

Table 3 lists the SEA values of BCC and HCirC lattice structures, and the data are extracted from the compression experiments of the resin samples and the samples after chemical plating and electroplating. The results of the BCC and HCirC lattice structures are compared, of which the data of HCirC lattice structure are obtained from two loading directions (e.g., *x*-axis and *z*-axis).

**Figure 12:**
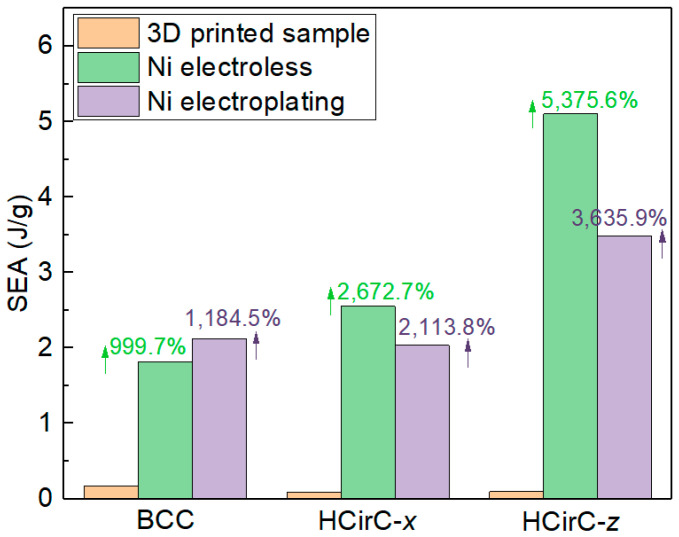
Comparison of the specific energy absorption between resin samples and the chemical plating and electroplating samples for BCC and HCirC lattice structures.

In Table 3 and Figure 12, for the BCC lattice structure, the lattice sample after electroplating shows the highest SEA values. However, for the HCirC lattice structure, the SEA values after chemical plating are higher than those after electroplating, for both loading directions. For the BCC lattice structure, the enhancement in SEA after chemical plating and electroplating is 999.7% and 1184.5%, respectively. For the HCirC lattice structure, the enhancement in SEA after chemical plating is 2672.7% (loading along the *x*-axis) and 5375.6% (loading along the *z*-axis), while the enhancement after electroplating is 2113.8% (loading along the *x*-axis) and 3635.9% (loading along the *z*-axis). Overall, both chemical plating and electroplating significantly enhance the SEA values of lattice structures, with respect to the resin samples. In addition, the HCirC is the bio-inspired hierarchical lattice structure, and its resin samples possess higher mechanical performance with respect to the simple BCC resin lattice structure. As can be seen from Table 3 and Figure 12, under the same loading direction and coating method, the HCirC lattice structure exhibits a higher overall energy absorption capacity than BCC. The results demonstrate that the combination of bioinspired unit cell design and metal plating can benefit the enhancement in the energy absorption capability of lattice structures.

### 3.4. Effect of Electroplating Time on the Mechanical Properties

**Figure 13:**
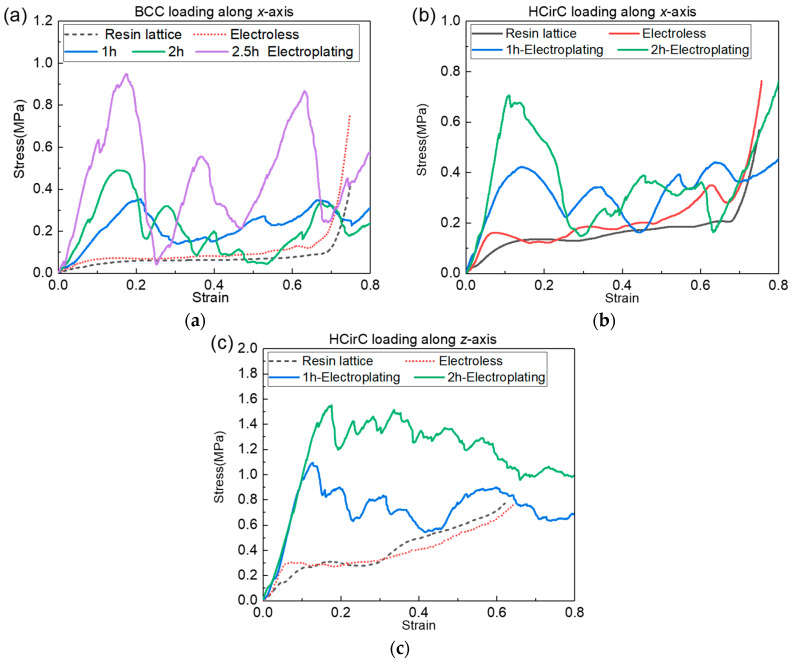
Effect of electroplating time on the mechanical properties: (**a**) BCC; (**b**) HCirC along *x*-axis; and (**c**) HCirC along *z*-axis.

In order to further investigate the effect of electroplating time on the mechanical properties of lattice structures, further metal plating experiments were conducted on BCC and HCirC lattice structures. With consistent control of the chemical plating time, experiments were performed on samples that underwent different electroplating times. The electroplating times for the BCC structure were controlled at 1 h, 2 h, and over 2.5 h, while for the HCirC structure, the electroplating times were 1 h and 2 h. After the electroplating experiments, the samples were cleaned and dried. The uniaxial compression tests were performed on the samples with different plating times using a universal testing machine, and the experimental data were recorded. Subsequently, the experimental data were processed, and stress–strain curves were plotted. Figure 13a shows the compression curves of the BCC lattice structure before and after chemical plating and the compression curves using different electroplating time values. Figure 13b,c, respectively, show the compression curves of the HCirC lattice structure along the *x*-axis and *z*-axis loading directions, before and after chemical plating, as well as the different electroplating times.

In Figure 13, it can be seen that the BCC structure exhibits a noticeable enhancement in stiffness and strength after electroplating for 2.5 h, compared to those after electroplating for 1 h and 2 h. In addition, it seems that the longer the plating time, the greater the oscillation amplitude of the compression stress. For the HCirC structure (Figure 13b,c), electroplating for 2 h shows a significant improvement in yield strength compared to that after electroplating for 1 h, but the enhancement in stiffness is not significant. It is noted that two nickel-coating methods were considered in the experiment. The first method is the chemical plating method. The second method is the composite electroplating method (e.g., electroplating after chemical plating). Electroplating is usually considered as an effective way to improve the coating thickness, after the chemical plating, and this has been used in many previous works with the same aims and scopes [44,52], although only using the chemical plating can also give relatively high coating thicknesses. In this study, the coating thickness of the composite electroplating is higher than that of the chemical plating, because the electroplating is performed after chemical plating. The coating thickness is the main factor that causes the enhancement in mechanical properties of lattice samples. The coating thickness can be controlled by many factors, such as coating time, the concentration of the plating solution, the current magnitude, and so on. In this study, the coating time is employed to investigate the influence of coating time on the mechanical performance of the lattice structure. The coating thickness will increase with the increase in coating time, thus bringing enhanced mechanical properties. The accurate control of coating thickness will be further investigated in our future work. Although the nickel coating on the lattice structure has been reported in many previous works, their study only considered the coating of some basic lattice configurations, such as BCC [53], OCT [32], and diamond unit cells [44]. In the present work, the chemical plating and electroplating were performed on the 3D-printed resin lattice samples. The nickel coating of bio-inspired hierarchical circular cell configuration is considered, the mechanical properties were compared with the BCC lattice structure, and the relevant deformation mechanisms were revealed. Overall, compared to chemical plating, electroplating with higher coating thickness has a more pronounced effect on improving the mechanical performance of lattice structures, and the mechanical performance of the lattice structure will increase with the increase in electroplating time.

## 4. Conclusions

In this study, two different lattice structures, the body-centered (BCC) and bio-inspired hierarchical circular (HCirC), were considered in the 3D printing, chemical plating, and electroplating experiments. Uniaxial compression experiments were performed, and the compression behaviors of two lattice structures under different metal plating methods were compared. The main conclusion is summarized as follows: (1) Both electroplating and chemical plating can significantly enhance the mechanical performance of lattice structures, with respect to their corresponding resin lattice samples. In addition, the electroplating generally shows better enhancement effects than pure chemical plating. (2) The shell–core structure formed by the metal composite coating causes changes of the deformation model from bending- to stretching-dominated, as well as the enhancement in mechanical properties. (3) By adopting a bioinspired unit cell design and combining the advanced coating technologies, the performance enhancement space of lattice metamaterials can be further improved. (4) Both electroplating and chemical plating improve the energy absorption capacity within the lattice structure compared to the polymer lattice template. It is worth noting that the metal coatings greatly increase the specific energy absorption (SEA) values of both chemical plating and electroplating. (5) The electroplating shows a more significant improvement in the mechanical performance of lattice structures, with respect to the chemical plating, and the longer the electroplating time, the stronger the mechanical performance of the lattice structure.

## Figures and Tables

**Table 1:** The mass of the fabricated lattice samples before and after nickel coating process (unit: g).

Lattice Structure	Resin Lattice	Electroless Plating Lattice	Electroplating Lattice
BCC	0.416	0.593	2.180
Mass of nickel (BCC)	-	0.177	1.764
HCirC	0.737	1.003	2.865
Mass of nickel (HCirC)	-	0.266	2.128

**Table 2:** The comparison of compression mechanical properties of BCC and HcirC before and after nickel-coating.

	Stiffness (Mpa)	Yield Strength (Mpa)	Specific Stiffness (Mpa∙cm^3^/g)	Specific Strength (Mpa∙cm^3^/g)
	BCC			
Resin sample	0.325	0.060	3.203	0.592
Electroless plating	3.025	0.210	20.895	1.448
Electroplating	50.314	2.694	94.534	5.063
	HcirC-*x* (Loading along *x*-axis)			
Resin sample	1.256	0.126	6.981	0.698
Electroless plating	7.420	0.515	30.331	2.105
Electroplating	31.587	1.077	45.159	1.540
	HcirC-*z* (Loading along *z*-axis)			
Resin sample	2.916	0.262	16.207	1.458
Electroless plating	22.071	2.435	90.222	9.952
Electroplating	77.896	4.528	111.365	6.474

**Table 3:** The comparison of energy absorption performance before and after metal plating for the lattice structures (unit: J/g).

	Resin Lattice	After Electro Less Plating	After Electro Plating
BCC	0.165	1.818	2.123
HCirC (*x*-axis)	0.092	2.551	2.037
HCirC (*z*-axis)	0.093	5.103	3.482

## Data Availability

The data that support the findings of this study are available from the corresponding author Mingzhi Wang, upon reasonable request.
